# The Role of Vitamin C as Adjuvant Therapy in COVID-19

**DOI:** 10.7759/cureus.11779

**Published:** 2020-11-30

**Authors:** Poona Kumari, Suman Dembra, Pariya Dembra, Fnu Bhawna, Ambresha Gul, Basma Ali, Hamza Sohail, Besham Kumar, Muhammad Khizar Memon, Amber Rizwan

**Affiliations:** 1 Internal Medicine, Ghulam Muhammad Mahar Medical College, Sukkur, PAK; 2 Medicine, Peoples University of Medical and Health Sciences for Women, Nawabshah, PAK; 3 Medicine, Jinnah Sindh Medical University, Karachi, PAK; 4 Internal Medicine, Jinnah Sindh Medical University, Karachi, PAK; 5 Internal Medicine, Qatar Hospital, Karachi, PAK; 6 Internal Medicine, People's University of Medical and Health Sciences for Women, Nawabshah, PAK; 7 Internal Medicine, Jinnah Postgraduate Medical Centre, Karachi, PAK; 8 Internal Medicine, Liaquat University of Medical and Health Sciences, Hyderabad, PAK; 9 Family Medicine, Jinnah Postgraduate Medical Centre, Karachi, PAK

**Keywords:** intravenous vitamin c, covid-19, pakistan

## Abstract

Background and objective

The anti-inflammatory properties of vitamin C (VC) and the promising results it has shown in the treatment for common cold have prompted clinicians to use it as adjuvant therapy in the treatment of COVID-19. The purpose of this study was to find out the role of VC as adjunctive therapy in coronavirus disease 2019 (COVID-19).

Methodology

This study was conducted from March to July 2020 in the COVID-19 unit of a tertiary care hospital in Karachi. In this randomized controlled trial (RCT), one group received the intervention [50 mg/kg/day of intravenous (IV) VC] along with the standard therapy, and the other group received standard therapy only. Data such as age, gender, vitals, and biochemical values as well as outcomes including the number of days required for treatment, hospital stay, need for ventilation, and mortality were compared between the two groups and recorded using a self-structured questionnaire.

Results

COVID-19 patients who received IV VC became symptom-free earlier (7.1 ± 1.8 vs. 9.6 ± 2.1 days, p-value: <0.0001) and spent fewer days in the hospital (8.1 ± 1.8 vs. 10.7 ± 2.2 days, p-value: <0.0001) compared to those who received standard therapy only. However, there was no significant difference in the need for mechanical ventilation (p-value: 0.406) and mortality (p-value: 0.31) between the two groups.

Conclusion

VC can significantly improve clinical symptoms in patients affected with COVID-19; however, it had no impact on mortality and the need for mechanical ventilation. More large-scale studies are required to further assess the role of VC in the treatment of COVID-19.

## Introduction

Coronavirus disease 2019 (COVID-19) is an infectious disease caused by a newly discovered strain of coronavirus called severe acute respiratory syndrome coronavirus 2 (SARS-CoV-2). Most infected people will develop mild to moderate illnesses with the most common symptoms being fever, dry cough, and fatigue, and recover without hospitalization [1]. Vitamin C (VC), otherwise known as ascorbic acid, is known to boost immunity and acts as a potent antioxidant. Therefore, VC can be beneficial in resolving infection and inflammation. A randomized controlled trial (RCT) has demonstrated a measurable benefit of VC supplementation in reducing cold episodes in young men with low to average VC status [2]. In a recent meta-analysis, VC supplementation was shown to reduce serum C-reactive protein (CRP) levels, particularly in younger subjects with higher CRP baseline levels, at a lower dosage, and with intravenous (IV) administration [3].

Potential benefits of VC have sparked an interest regarding finding its use in the treatment of COVID-19. In a case series involving VC administration to COVID-19 patients, there was a significant decrease in inflammatory markers, indicating that the use of IV VC in patients with moderate to severe COVID-19 disease may be clinically feasible [4]. Administration of high doses of VC as a therapeutic agent can favorably impact severely ill COVID-19 patients with viral pneumonia and acute respiratory distress syndrome (ARDS) by decreasing inflammation, pathogen infectiveness, and virulence, and also by optimizing immune defense, reducing tissue and organ injuries, and improving the overall outcome of the disease [5].

During the ongoing global outbreak of COVID-19, there are many studies and trials underway to figure out the therapeutic role of VC in COVID-19 patients. Moreover, there is so much more yet to be learned about this vitamin itself. We believe that our study will contribute significantly towards the efforts to find out if VC could be beneficial as adjunctive therapy in the treatment of COVID-19.

## Materials and methods

This prospective, open-label RCT was conducted in the COVID-19 unit of a tertiary care hospital in Karachi, Pakistan. Data of patients who were admitted with severe COVID-19 infection from March to July 2020 were included in the study. Patients were diagnosed with severe COVID-19 based on the national health guidelines of Pakistan [6]. Patients who needed mechanical ventilation within 12 hours of admission were excluded from the study. All subjects provided informed consent for inclusion before they participated in the study.

Patients were randomized to the interventional arm or placebo arm using a randomizer software. The interventional arm received 50 mg/kg/day of IV VC in addition to standard therapy for COVID-19 infection. The placebo arm received only the standard therapy for COVID-19. Standard therapy included antipyretics, dexamethasone, and prophylactic antibiotics and was comparable between both groups. Data were compared between the patients who received IV VC vs. those who did not receive it. Patients' gender, age, and vitals and biochemical values such as respiratory rate, oxygen saturation, CRP, and lactate dehydrogenase (LDH) levels were recorded using a self-structured questionnaire. The number of days required for the disappearance of symptoms, number of days spent in the hospital, need for ventilation, and mortality were also noted and compared for both groups.

The collected data were analyzed using SPSS Statistics version 21.0 (IBM Corp, Armonk, NY). Mean and standard deviations (SD) were calculated for numerical data. Frequency and percentages were calculated for categorical data. Frequencies were compared using a chi-squared test. Independent t-test and chi-square test were used as appropriate. A p-value of less than 0.05 indicated that there was a significant difference between the two groups and the null hypothesis was void.

## Results

A total of 150 patients were included in the study; 75 of them were randomized to the interventional arm and received IV VC in addition to standard therapy for COVID-19 infection, and 75 were in the placebo group and received only standard care for COVID-19 infection. There were 99 (56.9%) males and 76 (43.1%) females in the study. The differences in age, respiratory rate, levels of CRP, and LDH levels between the two groups were not statistically significant (Table 1).

**Table 1:** Comparison of demographics and clinical characteristics between the two groups CRP: C-reactive protein; LDH: lactate dehydrogenase; SOC: standard of care; SD: standard deviation; IV: intravenous

Characteristics	SOC + IV vitamin C (n=75), mean + SD	SOC without IV vitamin C (n=75), mean + SD	P-value
Age (in years)	52 ± 11	53 ± 12	0.59
Respiratory rate (BPM)	30.2 ± 5.7	29.8 ± 4.9	0.64
CRP (mg/L)	118.2 ± 16.2	116.2 ± 17.2	0.46
LDH (IU)	315.2 ± 87.6	311.3 ± 89.3	0.78
Oxygen saturation (%)	87.2 ± 4.6	86.1 ± 4.9	0.15

COVID-19 patients who received IV VC became symptom-free earlier (7.1 ± 1.8 vs. 9.6 ± 2.1 days, p-value: <0.0001) and spent fewer days in the hospital (8.1 ± 1.8 vs. 10.7 ± 2.2 days, p-value: <0.0001) compared to those who received standard therapy only. However, the overall difference regarding the need for mechanical ventilation and mortality between the interventional arm and the placebo group was not statistically significant (Table 2).

**Table 2:** Comparison of outcomes between the two groups *Statistically significant; **Statistically not significant SOC: standard of care; SD: standard deviation; IV: intravenous

Outcome	SOC + IV vitamin C (n=75)	SOC without IV vitamin C (n=75)	P-value
Days to be symptom-free, mean + SD	7.1 ± 1.8	9.6 ± 2.1	<0.0001*
Days spent in the hospital, mean + SD	8.1 ± 1.8	10.7 ± 2.2	<0.0001*
Need for mechanical ventilation, n (%)	12 (16%)	15 (20%)	0.406**
Overall death, n (%)	7 (9.3%)	11 (14.6%)	0.31**

## Discussion

SARS-CoV-2 primarily targets the respiratory system, leading to the development of severe ARDS and even respiratory failure. Symptoms manifested depend on the organ system involved and range from high-grade fever, dry cough, shortness of breath, sore throat, and fatigue to diarrhea, confusion, seizures, and impairment of taste and smell [7].

The infection caused by SARS-CoV-2 and its progression to respiratory failure is driven by a strong and dysregulated immune-inflammatory response, which leads to elevated levels of pro-inflammatory cytokines including interleukin 6 (IL-6) and endothelin-1 (ET-1) in the body and a “cytokine storm” causing accumulation of neutrophils within the lungs, destroying alveolar capillaries. VC exerts its effects by reducing the secretion of these pro-inflammatory cytokines from the immune effector cells and preventing neutrophils activation and accumulation and the formation of neutrophil extracellular traps, which is a biological event of alveolar vascular injury caused by neutrophil activation [8,9]. In China, 50 patients with moderate to severe COVID-19 were given 10-20 g/day of VC. The oxygenation index of the patients improved, and all the patients were eventually cured and discharged [10]. An RCT conducted in the United States (US) showed that administration of ~15 g/day of IV VC for four days may decrease mortality in patients with sepsis and ARDS [11]. In a recent meta-analysis of nine RCTs, 0.7-0.8 g/day dose of VC improved symptoms and reduced the duration of infection and time of indoor confinement in patients with common cold virus infection [12]. In another meta-analysis of eight RCTs, supplementation of 0.5-2 g/day of VC in 3,153 patients reduced the duration of the upper respiratory tract infection by 1.6 days, suggesting its potential role in the treatment of COVID-19 [13]. However, very few studies have been conducted to correctly predict the role of VC as adjuvant therapy for COVID-19, but those conducted have shown promising results related to high doses of VC due to its favorable side-effect profile [8].

The present study was conducted to analyze the benefit of using VC as an adjuvant therapy to treat COVID-19 patients presenting to a tertiary care hospital in Pakistan. This study also concluded that patients who received IV VC along with standard therapy for SARS-CoV-2 infection recovered earlier (7.1 ± 1.8 vs. 9.6 ± 2.1 days, p-value: <0.0001) and spent fewer days in the hospital (8.1 ± 1.8 vs. 10.7 ± 2.2 days, p-value: <0.0001) when compared to patients who only received standard therapy for SARS-CoV-2 infection. However, the overall difference in the need for mechanical ventilation and mortality rate between them was not significant. This was in contrast with the meta-analysis by Hemilä et al., which reported that high doses of IV VC shortened the length of mechanical ventilation by 14% in critically ill patients, accompanied by a significant reduction in the mortality rate [14,15]. An RCT conducted on 56 critical COVID-19 patients concluded that the difference was not significant in invasive mechanical ventilation-free days in 28 days (IMVFD28) between groups that received high-dose IV VC and the group that received bacteriostatic water infusion. However, the study reported reduced 28-day mortality in severe COVID-19 patients who received high-dose IV VC [16].

This study, to the best of our knowledge, is the first study to be conducted in Pakistan to analyze the role of VC as an adjunct therapy for COVID-19 patients admitted to hospitals. However, the study has its limitations. Since it was a single-center study, the sample was not diverse and care should be taken when extrapolating the results on a larger scale.

## Conclusions

Based on our findings, VC can significantly improve clinical symptoms of patients affected with SARS-CoV-2 and reduce days spent in the hospital; however, VC supplementation had no impact on mortality and the need for mechanical ventilation. Nevertheless, VC has been proven to improve immunity in various forms of virus infections, and more studies on a larger scale are needed to further assess the role of VC in the treatment of COVID-19.
